# Open Science Training in TRIPLE

**DOI:** 10.12688/openreseurope.15430.2

**Published:** 2023-06-05

**Authors:** Lottie Provost, Francesca Di Donato, Erzsébet Tóth-Czifra, Suzanne Dumouchel, Emilie Blotière, Yin Chen

**Affiliations:** 1 Istituto di Linguistica Computazionale “A. Zampolli”, Consiglio Nazionale delle Ricerche (CNR), Pisa, 56124, Italy; 2 DARIAH Coordination Office Berlin c/o Centre Marc Bloch e.V., Berlin, 10117, Germany; 3 TGIR Huma-Num, CNRS, Aubervilliers, 93300, France; 4 EGI Foundation, Amsterdam, 1098XG, The Netherlands

**Keywords:** Open Science, Training and education, FAIR data, EOSC, Scholarly practice, Project management

## Abstract

This case study focuses on the online training activities on Open Science delivered within the H2020 project Transforming Research through Innovative Practices for Linked Interdisciplinary Exploration (TRIPLE, Grant Agreement 863420). The project is dedicated to building a discovery platform for the Social Sciences and Humanities (SSH) and is committed to promoting and supporting the uptake of Open Science within research practices.

In order to address SSH research and training communities’ needs for enhanced competencies on Open Science and for stronger support in the Findable, Accessible, Interoperable, Reusable (FAIR) management of digital training materials, two reusable outputs were produced. The work carried out is presented as a novel approach to tackle the issues related to FAIRifying research and training practices and to create training resources whose reusability and relevance reaches beyond the project lifetime and framework. The case study presents the methods by which the results were produced so as to encourage and enable their future adaptation and reuse.

The TRIPLE Open Science training series (result 1) targets SSH researchers, research support personnel and infrastructure developers in need of practical tools and specific skills to integrate Open Science practices in their workflows. The training series provides 12 competence-oriented online training events in Open Access whose training materials are available as Open Educational Resources (OER).

The TRIPLE Training Toolkit (result 2) targets training organisers and research performing organisations who wish to design and manage training events as OERs and increase the impact of their training following good practice. The Toolkit is an easily reproducible workflow designed to help trainers minimise the time they spend in managing training events following FAIR practice. The workflow follows a FAIR-by-design method to address the frequent findability and reusability issues related to the management of digital training resources **.**

## Plain Language Summary

This case study presents the work carried out in the Transforming Research through Innovative Practices for Linked Interdisciplinary Exploration (TRIPLE) Project in the Task on Open Science training and guidelines. We produced two results for researchers and training organisers to reuse in their activities. The general objective is to provide them with knowledge, skills, tools and solutions that will help improve their research and training practices.

The first result is an online series of training events on Open Science topics. The training sessions are open to everyone and the slides and presentations of each event are available and can be reproduced.

The second result is a step-by-step process to organise training events following best practice. This process is made to be reused and adapted by training organisers for their activities.

## 1. Introduction

While in recent years research communities have witnessed a change in how science is conceived and practised ( Agreement on Reforming Research Assessment, 2022; Burgelman *et al.*, 2019; Deniz Beyan *et al.*, 2020), it is yet too early to celebrate the wide scale adoption of Open Science practices across countries, disciplines and career levels. The COVID-19 pandemic acted as a catalyst in transforming the way research is performed with a paradigm shift in how learners across the world access learning ( UNESCO, 2020). The situation also highlighted the need to upskill researchers in Findable, Accessible, Interoperable, Reusable (FAIR) ( Wilkinson *et al.*, 2016) and Open Science practices and to address the lack of FAIRness of training resources on the subject ( Directorate-General for Research and Innovation (European Commission), EOSC Executive Board, Manola N, *et al.*, 2021).

In the context of increasing availability of online training resources, the report Digital Skills for FAIR and Open Science ( Directorate-General for Research and Innovation (European Commission), EOSC Executive Board, Manola N, *et al.*, 2021) highlighted the importance of delivering sustainable, FAIR and qualitative training resources and ensuring they are made available as Open Educational Resources ^1^ (OER) to enable their reuse and adoption by others.

Among the identified constraints to a widespread uptake of Open Science practices in research workflows, it has been observed that the generic Open Science frameworks and solutions do not always fit well to the specific needs, workflows, data types and community practices of the diverse epistemic cultures across but even within certain research domains, such as the Social Sciences and Humanities (SSH) ( Deniz Beyan *et al.*, 2020; Tóth-Czifra, 2019). Successful implementation of such practices also takes place in specific disciplinary and geographical contexts ( Gotz *et al.*, 2020).

### 1.1. Methods

The TRIPLE consortium has been addressing these issues early from the proposal phase of the project onwards. To foster the uptake of Open Science practices and to address the rather urgent need for a common understanding of recent European Open Science advancements, an entire Work Package (WP) was dedicated to training and guidelines on Open Science and the European Open Science Cloud (EOSC). Our Open Science training efforts started out as a mutual learning exercise, giving unique chances for the project partners to bring together a very diverse range of profiles and expertise including SSH scholars and SSH-specific research support personnel, Open Science experts, engineers and other infrastructure providers as well as expertise in European research policy advisory, and to foster synergies between them, resulting in a series of online training events. As a second step, and following TRIPLE’s commitment to openly share project outcomes, we did not only open up the training events to the general public but also put provisions and protocols in place to allow for the reuse of training materials and training organisation resources in other contexts as well.

To address recurring challenges SSH researchers and trainers face when wishing to implement FAIR and Open Science principles in their practices and to improve researchers’ access to qualitative content and resources, two reusable sets of training outputs were produced. Both were shared with the SSH community to showcase good practice, enable reuse of the results and allow trainers within projects to replicate and adapt the FAIR-by-design process to their training activities ^2^. The present case study provides guidance for beginners in open science training and support staff in research performing organisations to reproduce the experiment.

The first output is the TRIPLE Open Science training series, a series of 12 openly accessible online training sessions specifically designed to provide skills and competencies to SSH researchers on FAIR and Open Science practices. It is presented in section 2. The second output is the TRIPLE Training Toolkit ( Di Donato *et al.*, 2022), an open and reusable workflow containing resources for trainers to reproduce and adapt to different training contexts in the future. It encapsulates the process implemented in the TRIPLE Open Science training series and as such can be consulted, followed, adapted and reused to organise and deliver FAIR-by design online training events. The Toolkit is showcased in section 3.

The training was primarily targeted at TRIPLE project members in order to ensure they all gained competencies in Open Science practices and workflows. However, the topics being of high interest for researchers at all career stages, the events were opened to the whole SSH community. The aim of opening up the TRIPLE training sessions was also to engage a larger audience and consequently maximise the dissemination of knowledge and skills in Open Science practices and the EOSC.

The organisation of the training series also gave the opportunity to bring together various European Open Science training providers so as to collectively frame common standards and best practices that will foster the implementation of FAIR and Open Science practices in the field of Open Science training. Accordingly, the TRIPLE Open Science training series was developed in collaboration with some of the major actors in the field of Open Science and related training such as CLARIN ERIC, the OpenAIRE Community of Practice for Training Coordinators, DARIAH ERIC and the SSHOC Training Community, which is an output of the SSHOC project, the SSH cluster of the EOSC.

The results presented in the following sections focus on the delivery of competence-oriented ^3^ training, publishing training materials as OERs and enabling trainers in the SSH community to reproduce the case study that is showcased following best practice.

## 2. From online training on Open Science to the design and delivery of OERs: the TRIPLE Open Science Training Series

### 2.1 Competence-oriented training

Between March 2021 and June 2022, 12 online training events were held with a total of 600 attendees ^4^ with the guiding objective to improve participants’ knowledge and skills on Open Science and the EOSC. All the information related to the Training series and individual events is available in the Training section of the TRIPLE website. To ensure long term access the materials are deposited in Zenodo under the OPERAS community.

This section details how we selected the training topics, defined learning outcomes and carried out the training assessment.

***Selection of topics for training events*.** A survey ^5^ was handed out twice to the TRIPLE Consortium (in December 2019, during the first phase of the project, and in December 2021) to define which topics project partners considered to be the most useful to develop skills and knowledge in relation to Open Science and the EOSC, and for which dedicated training could be helpful to their work. Based on two classifications, the survey suggested a list of 14 topics focusing on Open Science practices and skills, and on specific TRIPLE activities. We relied on the Open Science Taxonomy ( Pontika & Knoth, 2015), which represents the wide array of principles and practices encompassed under the term Open Science, and on the four Open skills categories outlined by the Expert Group on Education and Skills under Open Science ( Directorate-General for Research and Innovation (European Commission), O'Carroll C, *et al.*, 2017):

- Skills and expertise necessary for open access publications;
- Skills and expertise regarding research data and open access, data production, management, analysis/use/reuse, dissemination;
- Skills and expertise to act beyond their own scholarly and disciplinary community;
- Skills and expertise resulting from a general and broad concept of citizen science.

Following the answers to the survey, the training events addressed a range of topics covering Open Science research practices, current EOSC developments and GoTriple Innovative Services. To ensure high-quality training, we relied on the project partners’ internal competencies and skills and on external speakers’ expertise for specific topics.

The list of training events is reported in Table 1 with the respective area of competency (Open Science, EOSC, GoTriple).

**Table 1.** TRIPLE Open Science training events and related area of competency. TRIPLE, Transforming Research through Innovative Practices for Linked Interdisciplinary Exploration; EOSC, European Open Science Cloud; FAIR, Findable, Accessible, Interoperable, Reusable.

No.	Title of the training	Area of competency
1	CLARIN Café on the Rights of Data Subjects in Language Resources	Open Science
2	The Open Access Publishing Platform Open Research Europe	Open Science
3	EOSC Onboarding	EOSC
4	EOSC - State of the Art and Perspectives	EOSC
5	FAIR Data in the Social Sciences and Humanities	Open Science
6	EOSC Architecture	EOSC
7	Visual Data Discovery for Social Sciences and Humanities	GoTriple
8	The Importance of User-Centred Design for Open Science	Open Science
9	The GoTriple Trust Building System	GoTriple
10	Multilingual Vocabularies for Social Sciences and Humanities	Open Science
11	The GoTriple Pundit Annotation Tool	GoTriple
12	Copyright and Academia in the Digital Era	Open Science

***Learning outcomes*.** Consideration was also given to ensuring that the objectives of the training had been met and that learners successfully acquired new skills and competencies. To this effect, learning outcomes were also defined in the design phase of the training events. Learning outcomes are “statements that describe or list measurable and essential mastered-content knowledge - reflecting skills, competencies and knowledge that students have achieved and can demonstrate upon successfully completing a course” ( Bezjak *et al.*, 2018).

In order to help learners assess the relevance of the training for their needs and therefore to maximise the impact of the training itself, each training event was promoted and shared with the community with a clear list of learning outcomes. The EOSC Synergy Online Training Handbook ( Clare *et al.*, 2022) proved to be helpful in this part of the process, particularly the Initial training analysis worksheet, which includes a section on benefits and outcomes for learners.

Table 2 references the learning outcomes for each training event. Measurable performance verbs were used to emphasise which new skills and competencies learners will acquire after watching the training event. The learning outcomes listed below are intended to complete the following statement: “After watching this training you should be able to …”.

**Table 2.** Learning outcomes for TRIPLE Open Science training events. TRIPLE, Transforming Research through Innovative Practices for Linked Interdisciplinary Exploration; EOSC, European Open Science Cloud; FAIR, Findable, Accessible, Interoperable, Reusable; SSH, Social Sciences and Humanities.

No.	Title of the training
1	**CLARIN Café on the Rights of Data Subjects in Language Resources**
Learning outcomes	● Explain what rights the GDPR grants to data subjects. ● Identify if these rights are limited when personal data is processed for the purpose of language research. ● Identify who is responsible for handling data subjects’ requests related to the exercise of their rights.
2	**The Open Access Publishing Platform Open Research Europe (ORE)**
Learning outcomes	● Explain what the ORE publishing platform is. ● Identify the benefits the ORE platform provides to researchers. ● Use the ORE platform in detail. ● Discuss how the ORE platform will facilitate compliance to European Open Access terms of funding.
3	**EOSC Onboarding**
Learning outcomes	● Explain the minimum criteria to become a provider and the requirements to onboard services into the EOSC Portal. ● Apply the process of onboarding services into the EOSC Portal. ● Gather information from the TRIPLE project to be taken into account for the next iteration of the Portal development.
4	**EOSC - State of the Art and Perspectives**
Learning outcomes	● Explain the latest stages of the EOSC development. ● Describe the recent changes in the EOSC governance. ● Identify the next steps for the EOSC implementation. ● Contribute to the EOSC.
5	**FAIR Data in the Social Sciences and Humanities**
Learning outcomes	● Describe how research data is defined in SSH. ● Demonstrate the importance of FAIR principles in the management of research data in SSH. ● Apply FAIR principles to data and implement them in SSH.
6	**EOSC Architecture**
Learning outcomes	● Explain the principles of the EOSC Architecture. ● Describe the main components of the EOSC Architecture. ● Analyse interoperability issues in the EOSC Architecture. ● Name the projects and further developments of the EOSC Architecture.
7	**Visual Data Discovery for Social Sciences and Humanities**
Learning outcomes	● Identify ways in which knowledge maps and streamgraphs can help researchers overcome discovery challenges. ● Visually explore research topics and recognize trends in research with GoTriple. ● Produce and interpret knowledge maps and streamgraphs on the GoTriple platform.
8	**The Importance of User-Centred Design for Open Science**
Learning outcomes	● Understand the importance of user centred design in the Open Science perspective. ● Synthesise the main phases of the iterative design process. ● Define user requirements, scenarios and personas. ● Understand the main differences and complementarities between the cognitive walkthrough method and the artefacts ecology mapping method.
9	**The GoTriple Trust Building System**
Learning outcomes	● Create a profile on the Trust Building System (TBS) application. ● Post a request. ● Interact with peers (invitations, introductions, group creation).
10	**Multilingual Vocabularies for SSH**
Learning outcomes	● Explain what SSH vocabularies are and why they are so important. ● Describe how to create a multilingual SSH vocabulary. ● Identify management needs related to the large variety of vocabularies in the SSH. ● Summarise how to build an interoperable infrastructure for vocabularies.
11	**The GoTriple Pundit Annotation Tool**
Learning outcomes	● Understand the purposes and functionalities of the Pundit Annotation Tool. ● Use Pundit in the SSH research context.
12	**Copyright and Academia in the Digital Era**
Learning outcomes	● Understand what changed in the digital era regarding copyright. ● Summarise briefly international and European legislation. ● Explain the principles of ownership and economic exploitation of academic works.

***Training assessment*.** To measure the impact of the training on participants’ practices, a short post training survey ^6^ was handed out at the end of each training session. This method for training assessment was put in place in September 2021 and lasted until June 2022.

One of the risks of sharing surveys after a training event is to receive low feedback. To counter this possibility and to maximise the number of answers, a Mentimeter survey was showcased to participants before closing each training event.

**Figure 1.**
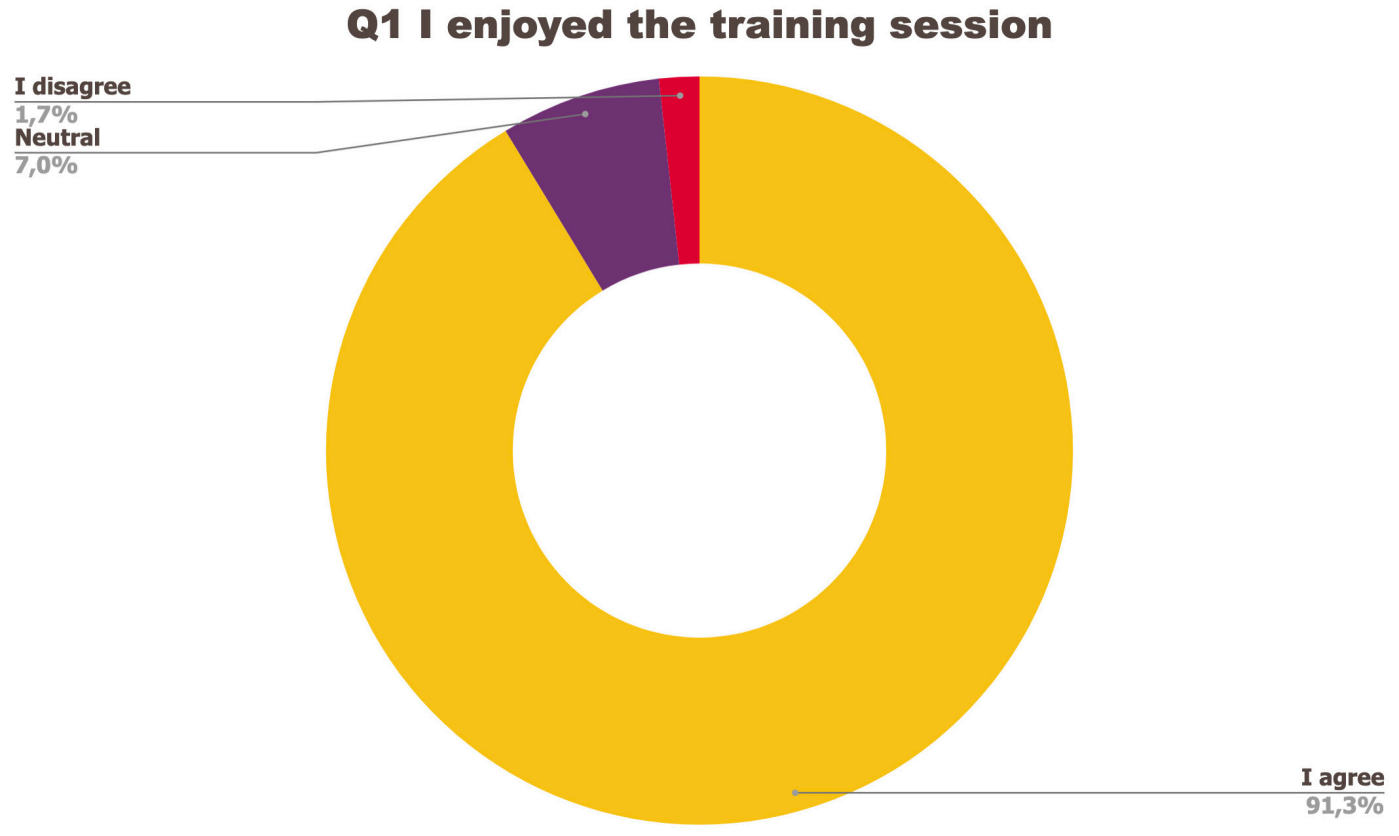
Summary of answers to Q1 “I enjoyed the training session”.

The survey consisted of a set of five questions regarding the organisation of the event to which participants could answer: I strongly agree; I agree; Neutral; I disagree; I strongly disagree. Figure 1 to Figure 5 below represent the aggregated answers to the survey for eight training events so as to outline the general performance of the training series between September 2021 and June 2022. For clarity purposes the answers “I strongly agree” and “I agree” were grouped together, the same was done for negative answers.

**Figure 2.**
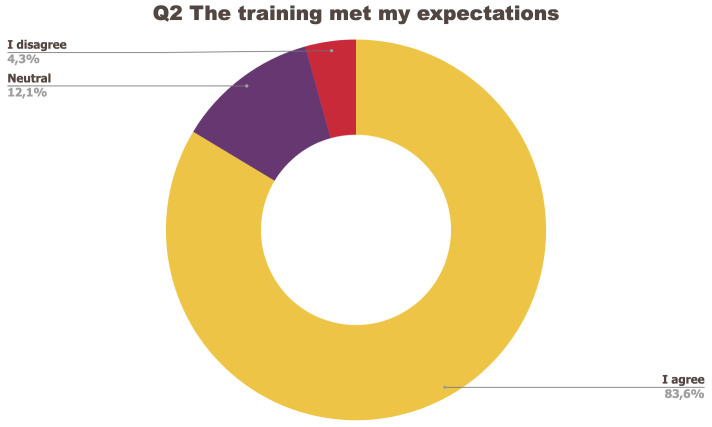
Summary of answers to Q2 “The training met my expectations”.

**Figure 3.**
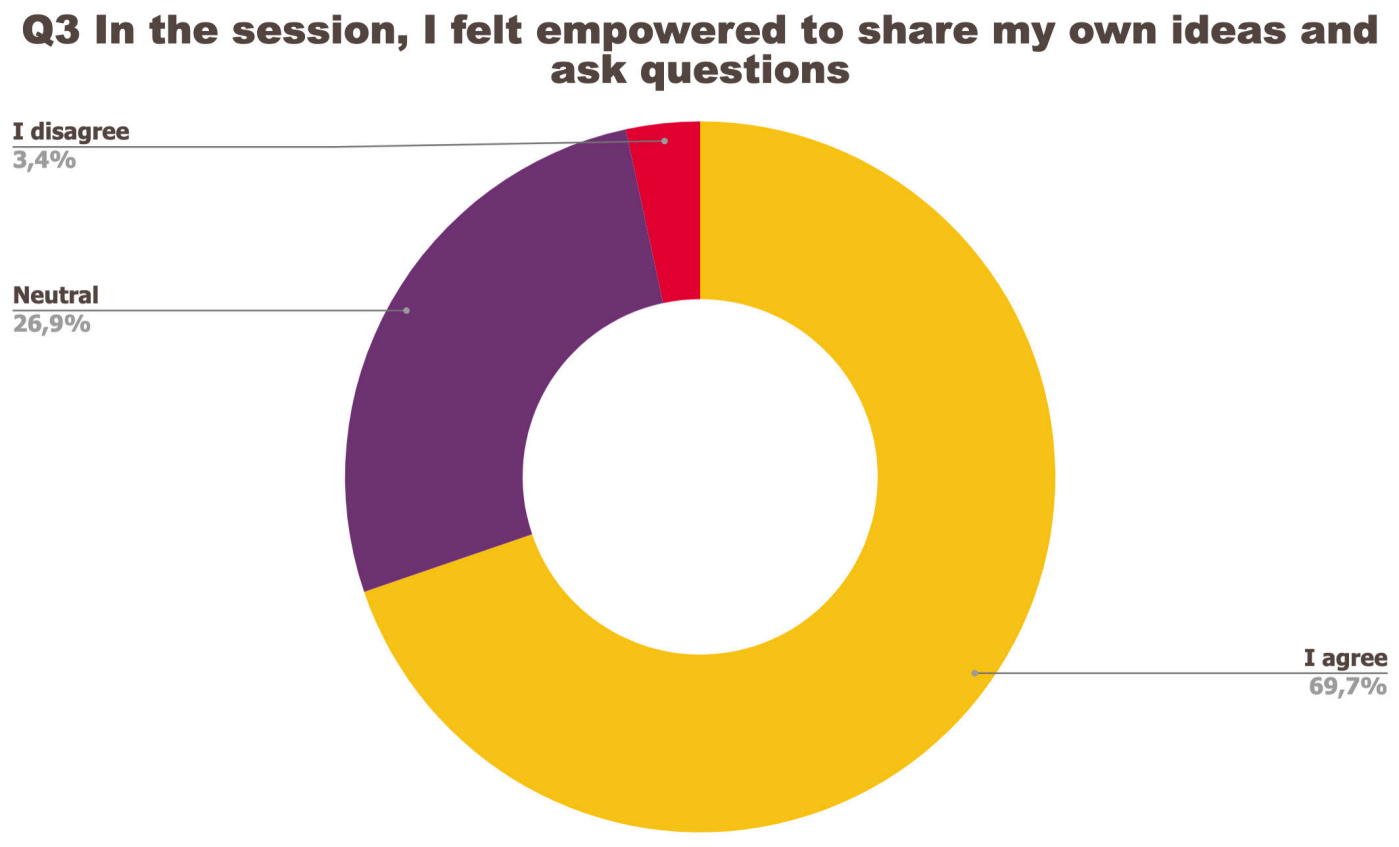
Summary of answers to Q3 “I felt empowered to share my ideas and ask questions”.

**Figure 4.**
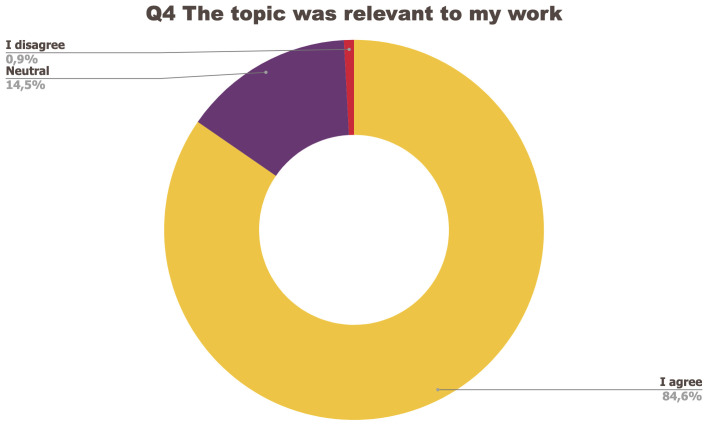
Summary of answers to Q4 “The topic was relevant to my work”.

**Figure 5.**
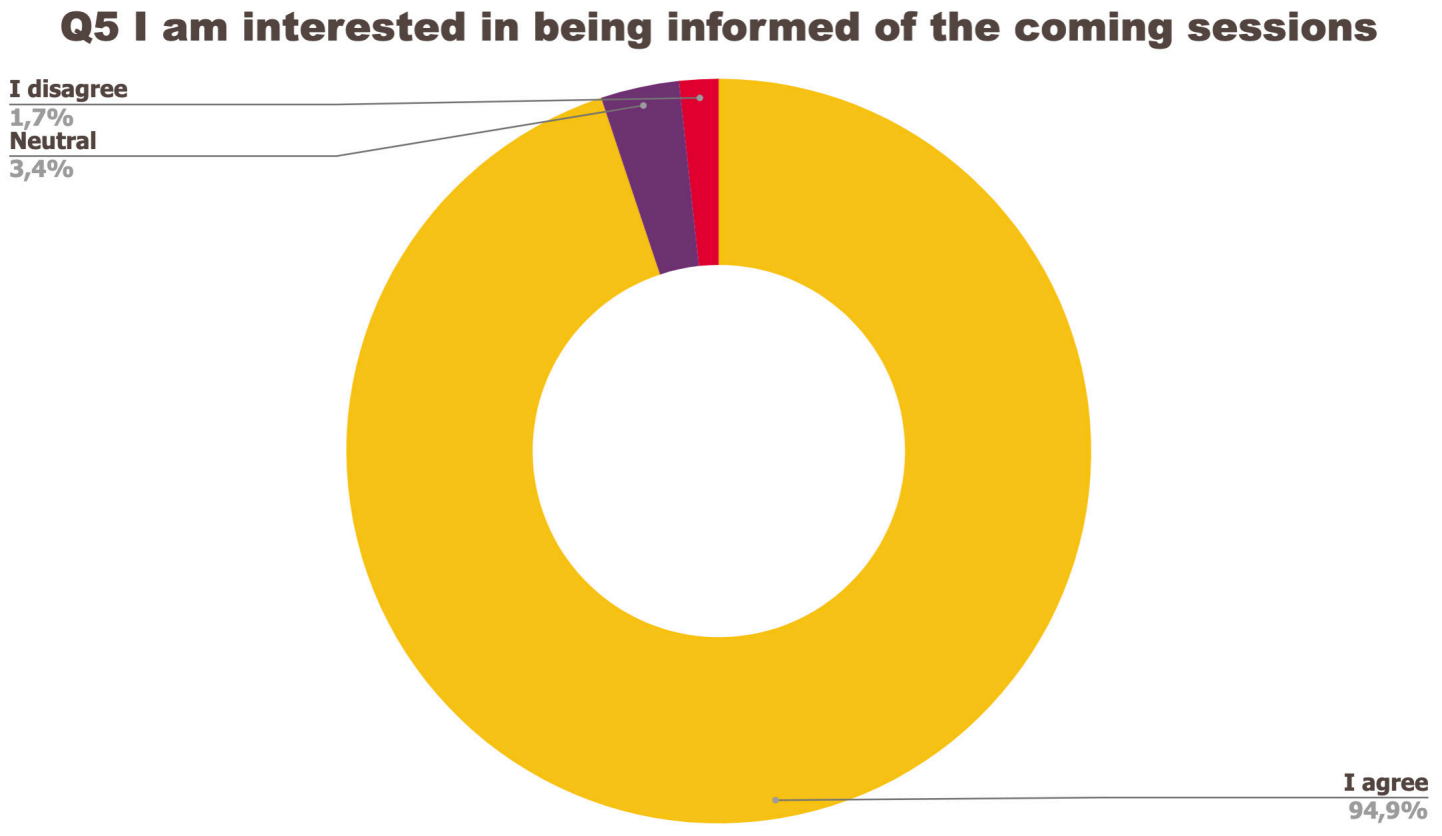
Summary of answers to Q5 “I am interested in being informed of the coming sessions”.

The results of the post-training survey underlined the successful achievements of the Training series: 91.3% of the respondents indicated that they enjoyed the training session (Q1) and 83.6% said the training met their expectations (Q2). Attendees’ positive response (84.6%) to the question “The topic was relevant to my work” (Q4) emphasises the realisation of one of the objectives that had been set for the Training series, which is to deliver training sessions that help attendees solve the problems they encounter in their daily research practices and provide them with new skills to tackle a number of specific issues. The attendees showed a high interest for future training sessions with 95% of the respondents who answered positively to this question (Q5). Q3 (“I felt empowered to share my own ideas and ask questions”) received the lowest positive response rate with just under 70% of positive feedback, and 26.9% neutral. Negative feedback was overall very low with 1 to 4% of respondents indicating they disagreed with the previous statements.

### 2.2 Open Educational Resources

Following the vision of the TRIPLE project to “improve SSH researchers’ access to content and resources”, we ensured that all training resources created within the project were in line with the FAIR principles, the final aim being to produce FAIR-by-design training materials. The process of providing training materials as OERs started with sharing and sustainability considerations in mind. This involved planning and investing resources not only in the documentation of the training materials themselves, but also in auxiliary materials which enable learners to follow and eventually reproduce the process of delivering training materials in a way that is based on Open Science practices. The various steps which led to sharing the training materials as OERs are detailed below.

All the training materials produced within the TRIPLE Open Science Training Series are deposited on a cross-domain repository such as Zenodo and on the SSH specific repository NAKALA. In order to foster the uptake of best practices within the SSH community particularly, the materials are uploaded on Zenodo under the OPERAS Community and can also be accessed by filtering the search by grant number (863420) in the repository. The video recordings are available on YouTube on the TRIPLE Project Channel. A persistent identifier (PID) is consistently attributed to each training resource to ensure they are permanently findable. Digital object identifiers (DOI) are used to identify training materials and Open Researcher and Contributor IDentifiers (ORCID) are used for authors. The training materials are shared under the licence Creative Commons Attribution International 4.0 (CC-BY-4.0) and access rules are consistently included in the metadata.

For interoperability purposes, the training materials are appropriately described with Zenodo's own standardised albeit generic metadata schema, which enables machine and human-reading. The metadata describing our training materials uses controlled vocabularies. When depositing on Zenodo, a dropdown list allows users to choose from a set of predefined values to describe the digital training material (document type, access rights, grant…). The materials are uploaded in .pptx and in .pdf formats to ensure permanent access, interoperability and eventual reuse and adaptation. Information on how to cite the training material is also part of the metadata as this helps make the material reusable for both trainees and trainers and enhances the redistribution of the training resources. Because the training series are project-based, no resources will be allocated to update the training materials after the end of the project. To counter this limitation, the timestamp of the last upload or update is always visible.

Table 3 below summarises the actions taken to manage training materials sustainably in the TRIPLE Open Science training series following the four aspects of FAIR. It is worth noting that the FAIR principles go into more detail than what is reported in Table 3. The aim of this table is not to reduce the complexity of the FAIR principles but rather to show beginners in the organisation of FAIR training events that following FAIR practices can be achieved starting from small steps.

**Table 3.** Simple steps to manage training materials following the FAIR principles. FAIR, Findable, Accessible, Interoperable, Reusable; PID, persistent identifier; DOI, digital object identifiers; ORCID, Open Researcher and Contributor IDentifiers.

FINDABLE	ACCESSIBLE	INTEROPERABLE	REUSABLE
Deposit training materials in Open Access in a trusted repository (Zenodo)	Licence the training under CC-BY-4.0	Use a standardised metadata schema such a Zenodo’s	Use an editable format such as .odp, .pptx
Assign a PID to the training: DOI for training materials, ORCID for authors	Include access rules as part of the metadata		Include information on how to cite the training as part of the metadata
			Always show the timestamp of the last upload or update of the training

To further disseminate the Training series and to increase discoverability possibilities beyond the scope of the TRIPLE project, the training materials are linked on the DARIAH-Campus platform ^7^ as external resources to which training-specific metadata ^8^ is added. The platform also enables one to explore training materials based on the context in which they were produced. All the TRIPLE Open Science training resources can therefore be found under the dedicated TRIPLE section. Each learning resource on DARIAH-Campus includes an introduction to the topic of the training, a set of clear learning outcomes, a video box directly linking to the video recording on YouTube and the DOI link to the training materials on Zenodo (slides, presentation). An important improvement to further enable accessibility would be to include HTML files so as to allow individuals with impaired vision to navigate the content effectively, especially when using screen readers.

Finally, to consolidate good practice with the creation of FAIR-by-design online training events and materials, the training materials (slides, video recordings) were made available along with the supplementary materials that guided the process. A sustainability document specifically dedicated to the design and delivery of online training materials and forming part of the project-level DMP details the protocol for the collection, use and storage of related data.

As presented above, the publication of training materials as OERs supports SSH researchers’ access to training materials all the while promoting good practice. The work carried out in this task also led to a reflection on the need to strengthen trainers' capacity to create, adopt and adapt OERs so as to fully integrate them in their training practices. The following section presents the outcome of this work: an adaptable workflow to design and deliver training events that follow the FAIR principles and publish training materials as OERs.

## 3. Achieving FAIR-by-design in the organisation of training events: the TRIPLE Training Toolkit

To facilitate the organisation of training events, a set of guidelines were created and shared internally ^9^. These guidelines were implemented among team members as a repeatable process to ensure a consistent application of Open practices within the development of the task and to guarantee a FAIR management of TRIPLE training materials.

The organisation and delivery of the training series led to a reflection on how to practically contribute to overcoming the challenges trainers face in the organisation of FAIR-by-design training events (i.e., training events that consider and implement the FAIR principles from the onset). To this end, the internal guidelines for organising training events were adapted into the TRIPLE Training Toolkit ( Di Donato *et al.*, 2022), to provide trainers with an open and reusable organisational process that is FAIR from the start and which can be adapted for each training event. Given the project-based context, trainers within projects are considered to be the best target group for using the Toolkit. However, the open workflow is generic and can also be adapted for beginners in open science training and support staff in research performing organisations who are involved in training activities.

When designing and delivering training events, we considered that trainers want to focus on producing impactful training that is visible to the community, reusable and reflects best practices. In order to spare time in the process, avoid double efforts and increase the reuse of existing training materials, trainers need a reproducible method they can adapt to their own training activities and implement easily.

Based on best practices, the Toolkit was framed to help trainers easily reproduce our workflow and manage training events and training materials sustainably. It maps the process of organising training events in five phases in which reference documents (11 in total) are indicated as reproducible templates for trainers to adapt. The files contained in the Toolkit are deposited in Zenodo in Open Access along with practical guidance on how to use them in order to enable replication of the process.

The following two subsections show how the Toolkit was adapted to trainers to make it more user-friendly and enable its reuse. Subsection 3.1 presents the process of organising training events, and subsection 3.2 explains the purpose of each reference document and how to reuse them. The aim is to make it as easy as possible for trainers to use and adapt the Toolkit so as to support the implementation of FAIR principles in their workflows and to enhance the visibility and impact of their training and materials.

### 3.1 An open workflow to navigate the process of organising FAIR training events

**Figure 6.**
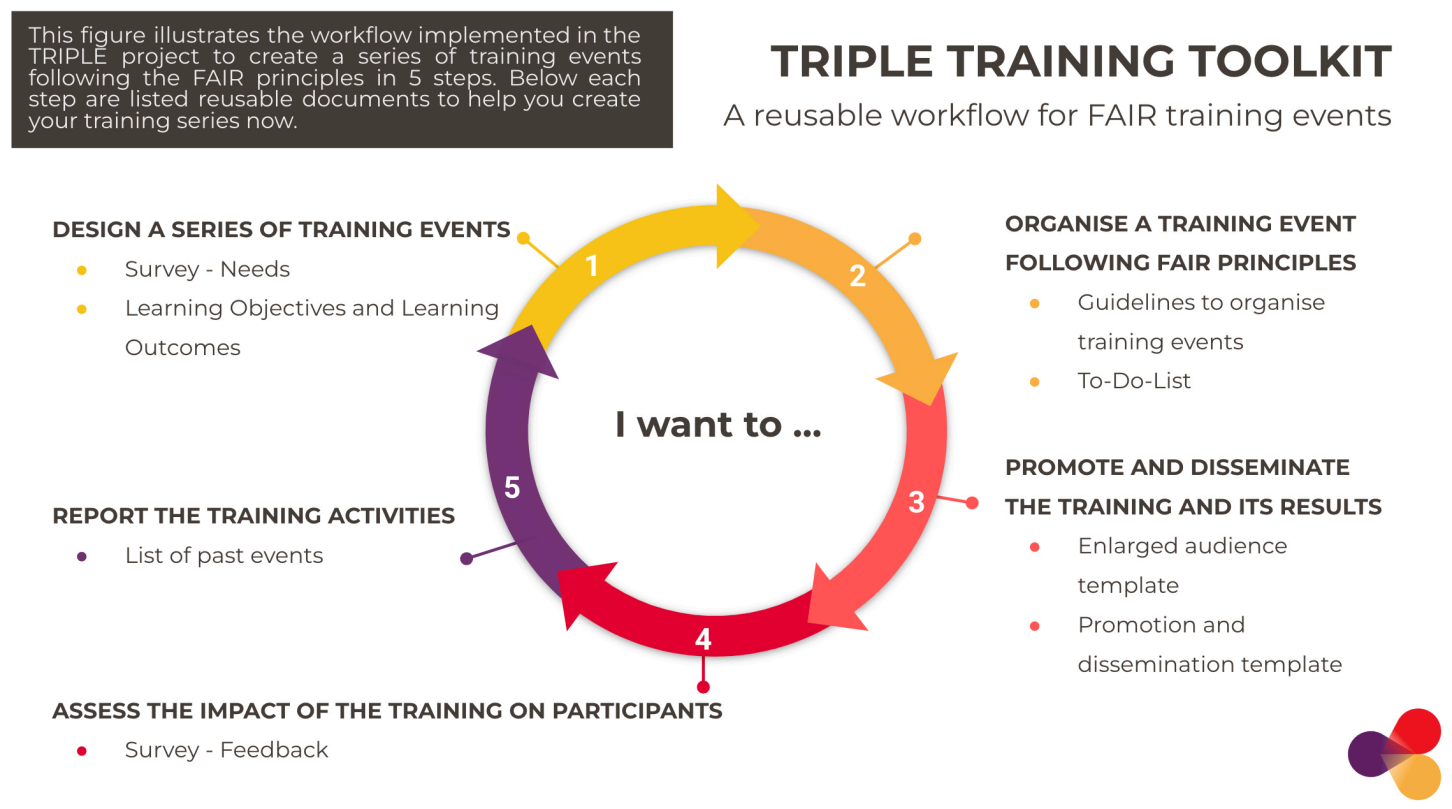
A workflow to organise training events in five steps with reusable reference documents. TRIPLE, Transforming Research through Innovative Practices for Linked Interdisciplinary Exploration; FAIR, Findable, Accessible, Interoperable, Reusable.

In order to successfully manage training events and materials following a FAIR-by-design process, trainers need to visualise what the task will include. Figure 6 was created with the purpose to help trainers visualise the entire process of designing and delivering FAIR training events following five phases. Following the process represented in Figure 6, training organisers can implement FAIR standards from the onset, minimise the time spent following best practice, all the while maximising the impact of the training materials through enhanced discoverability and reusability possibilities.

The process is shown as a circle that represents the user journey in organising a training event (or a series of events). Starting from the inside of the circle, “I want to…”, the training organiser is then directed towards a number that corresponds to a specific phase of the workflow. The phases are numbered following a chronological order of implementation; however, some phases may overlap (such as phase 2 and 3, which are usually carried out contemporaneously, or phase 5, which concerns the entire process). In each phase, reproducible reference documents are indicated; they serve as templates for users to duplicate and adapt to their training.

The five steps of the process are listed below. The reference documents in each step of the process are presented in section 3.2.

**1 Design a series of training events**

The design phase is intended as the preparatory phase in which the training needs of the target group are assessed to define relevant training topics and objectives. As presented in section 2, it includes the initial reflection on how to create impactful training and deliver new skills and competencies to participants. The activities in this phase can be reiterated throughout the training series to ensure the training is still adapted to evolving expectations.

In this phase, users can refer to two reference documents: 1) the template survey on training needs and 2) the example provided by the list of learning objectives and outcomes. Clearly setting training goals and identifying measurable criteria at the beginning of the course can help trainers be more focused on relevant training activities, leading to improvements in training efficiency.

**2 Organise a training event following the FAIR principles**

The organisation phase includes a set of activities aimed at successfully delivering the training event. In this phase, training organisers take care of various aspects: coordination and communication (with speakers, moderators and participants), logistical (location of the event, invitations, management of software/Zoom room), and reporting. Because this phase usually involves various actors (organisers, speakers, moderators, other team members), it requires a well-structured division of tasks to ensure that operations run smoothly.

In this phase, training organisers can rely on the internal guidelines and checklist instruments provided in the Toolkit. These reference documents will also help trainers ensure they consistently follow the FAIR principles and manage their training materials sustainably.

**3 Promote and disseminate the training and its results**

The promotion and dissemination phase takes place before and after the event. It includes the initial promotion activities aimed at increasing the training audience in numbers and diversity (in terms of field of work for example), which are carried out in the early stages of the preparation of the training series. Successive campaigns targeting specific groups or networks of interest are then carried out to promote each training event individually. The results of the training (materials, video recording) are finally disseminated in collaboration with relevant stakeholders.

In this phase, the reference documents will support trainers in reaching out to new stakeholders and keeping track of their audience growth and estimated outreach through promotion and dissemination activities. These are often required by funders to measure the impact of the activities.

**4 Assess the impact of the training on participants**

The impact of the training is assessed at the end of the event through an online survey instrument, which collects participants feedback using Mentimeter. The reason for assessment and the results of the post-training survey in the TRIPLE Training series have been presented in section 2.1.

In this phase, a template survey is provided to help training organisers create their own training assessment, ensure their objectives have been met and adapt the training according to the feedback they receive.

**5 Report on the training activities**

The last phase of the workflow covers the entire training series and focuses on reporting relevant information on past events and gathering them in one place. Consistent reporting activities help training organisers keep track of the work carried out, discuss improvement strategies, and avoid double efforts for future team members.

The list of past events provided in the Toolkit serves as an example for training organisers to reproduce to report on their training activities.

**Figure 7.**
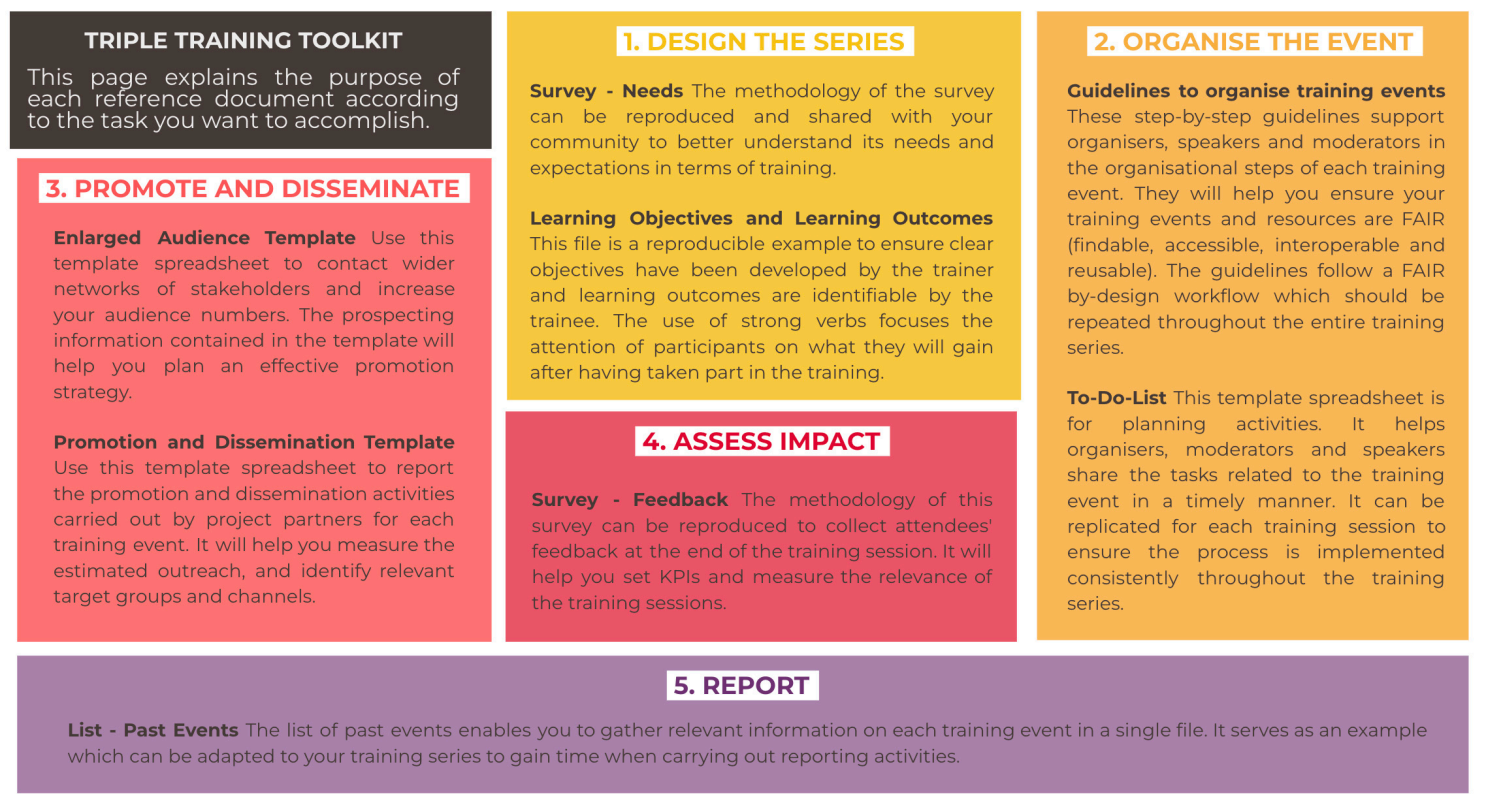
Purpose of the reference documents for each phase of the process. TRIPLE, Transforming Research through Innovative Practices for Linked Interdisciplinary Exploration; FAIR, Findable, Accessible, Interoperable, Reusable.

The purpose of each reference document is presented in Figure 7 in the following subsection.

### 3.2 How to reuse the reference documents in each phase of the process

The TRIPLE Training Toolkit takes into account the various needs trainers may have when it comes to applying Open Science and FAIR Data management practices to their training activities. While the previous subsection presented the user journey as a means to illustrate the generic process of delivering FAIR-by-design training events, Figure 7 below details the purpose of the reproducible templates in each step of the workflow with the aim to facilitate trainers’ reuse and adaptation of the Toolkit for their own training activities.

The reference documents indicated within the workflow are made to be reproduced and adapted and Figure 7 should enable training organisers to measure their relevance for their training activities. The purpose of each reference document as presented above is reported below ^10^.

There are two reference documents for Step 1 – Design the series:

- Survey - Needs. The methodology of this survey can be reproduced and shared with your community to better understand its needs and expectations in terms of training.
- Learning Objectives - Learning Outcomes. This file is a reproducible example to ensure clear objectives have been developed by the trainer and learning outcomes are identifiable by the trainee.

Two reference documents are helpful for Step 2 – Organise the Event:

- Guidelines to organise training events. These step-by-step guidelines support organisers, speakers and moderators in the organisational steps of each training event. They will help you ensure your training events and resources are FAIR (Findable, Accessible, Interoperable, Reusable). The guidelines follow a FAIR by-design workflow which should be repeated throughout the training series.
- To-Do List. This template spreadsheet is for planning activities. It helps organisers, moderators and speakers share the tasks related to the training event in a timely manner. It can be replicated for each training session to ensure the process is implemented consistently throughout the training series.

Two reference documents support Step 3 – Promote and Disseminate:

- Enlarged audience template. Use this template spreadsheet to contact wider networks of stakeholders and increase your audience numbers. The prospecting information contained in the template will help you plan an effective promotion strategy.
- Promotion and Dissemination template. Use this template spreadsheet to report the promotion and dissemination activities carried out by project partners for each training event. It will help you measure the estimated outreach and identify relevant target groups and channels.

One reference document is useful for Step 4 – Assess Impact:

- Survey - Feedback. The methodology of this survey can be reproduced to collect attendees' feedback at the end of the training session. It will help you set KPIs and measure the relevance of the training sessions.

Finally, one reference document is related to Step 5 – Report:

- List - Past events. The list of past events enables you to gather relevant information on each training event in a single file. It serves as an example that can be adapted to your training series to gain time when carrying out reporting activities.

## Conclusions

The TRIPLE Open Science training series has the potential to promote change in research practices through targeted training but also because it provides accompanying documentation, tools and good practices that contribute to building community standards and increase the reusability and reproducibility of open training content and strategy.

It is expected that the TRIPLE Open Science training series contribute to promoting Open Science practices by providing useful guidance about some specific tools developed or used within the TRIPLE project, that can be reused or adapted for other communities (such as the Pundit annotation tool, the Trust Building System, and the visualisation services). In this perspective, the training sessions are also relevant for the development of concrete Open Science services or to contribute to different interoperability issues.

The work in this case study supports researchers in the uptake of Open Science practices through the delivery of skill-oriented training events and by showcasing good practice in the management of training materials as OERs. Moreover, by openly sharing the training series and the reusable workflow implemented within the task, both researchers and trainers are provided with ready-to-reuse examples they can reproduce and adapt to organise training events and practically implement FAIR principles into their practices. In addition to providing online training, we contribute to strengthening networks and communities of practice by sharing our method and making it available for reproduction and adaptation.

Lastly, this training series is FAIR by design, which means it can be easily reused and adapted for further needs but it can be seen as a concrete example of the training methodology that can be used to ensure a proper uptake by the scientific community.

## Ethics and consent

This project has been ethically approved *via* the TRIPLE project coordinator. The training events were fully recorded in audio and video with the consent of the participants. Participants gave their informed written consent for the use and publication of their data.

## Data Availability

Zenodo: TRIPLE Training Toolkit (3.0). https://doi.org/10.5281/zenodo.6256197 ( Di Donato *et al.*, 2022). This project contains the following underlying data: 01 README_TRIPLE_Training_Toolkit. (A text file that details the contents and structure of the TRIPLE Training Toolkit.) 02 TRIPLE_Training_Toolkit_Workflow1. (An image that illustrates the user journey as a process to design a series of training events following the FAIR-by-design method.) 03 TRIPLE_Training_Toolkit_Workflow2. (An image that provides an explanation of the purpose of each reference document in the TRIPLE Training Toolkit according to the task the user wants to accomplish.) 04 Guidelines_Organisation_TRIPLE_Training_Toolkit. (A text file containing a practical step-by-step guide, which can be duplicated and repeated easily to support the organisers, moderators and speakers.) 05 To_Do_TRIPLE_Training_Toolkit. (A template spreadsheet to support organisers, moderators and speakers in sharing the tasks related to the training sessions and which gives a clear timeline of the actions to be performed.) 06 List_Past_Events_TRIPLE_Training_Toolkit. (A text file referencing all past TRIPLE training events with the name of the contributors, a short description of the event and the links to the resources.) 07 Training_Objectives_Learning_Outcomes_TRIPLE_Training_Toolkit. (A text file that provides an overview of the training objectives and learning outcomes for each training session.) 08 Internal_Training_Needs_Survey_TRIPLE_Training_Toolkit. (A text file containing the methodology of the survey, which was shared twice with the TRIPLE consortium to assess partners’ training needs.) 09a Internal_Training_Needs_Results1_TRIPLE_Training_Toolkit. (An image that shows the results of the first iteration of the questionnaire on internal training needs.) 09b Internal_Training_Needs_Results2_TRIPLE_Training_Toolkit. (An image that shows the results of the second iteration of the questionnaire on internal training needs.) 10 Post_Training_Survey_TRIPLE_Training_Toolkit. (A text file containing the methodology of the survey shared from September 2021 onwards at the end of the training sessions to collect attendees’ feedback and measure the relevance and utility of the training series.) 11 Post_Training_Survey_Results_TRIPLE_Training_Toolkit. (A spreadsheet containing the answers to the post training survey shared with training participants. The procedure was implemented in September 2021, the results therefore reflect the period from September 2021 to July 2022 and not before.) 12 Promotion_Dissemination_Template_TRIPLE_Training_Toolkit. (A template spreadsheet in which project partners report their promotion and dissemination activities regarding the TRIPLE training series.) 13 Enlarged_Audience_Template_TRIPLE_Training_Toolkit. (A template spreadsheet containing the prospecting information, which was collected to widen the audience of the TRIPLE training sessions.) Data are available under the terms of the Creative Commons Attribution 4.0 International license (CC-BY 4.0).
