# Supplementary material for: Open Science Training in TRIPLE
Source: Open Res Eur. 2023 Jun 5;3:39. Originally published 2023 Feb 23. [Version 2] doi: 10.12688/openreseurope.15430.2 (PMC10445817; doi:10.12688/openreseurope.15430.2)
Supplement: Peer review history

# Peer review: Reviewer response for version 1

**DOI:** 10.21956/openreseurope.16681.r31247

**Published:** 2023-04-24

**Authors:** Paola Masuzzo

**Affiliations:** 1 Institute for Globally Distributed Open Research and Education, Gothenburg, Sweden

This is a much needed and well written work providing both Open Education Resources for Open Science training, and freely reusable guidelines for organizing training sessions. Both these research outputs are extremely important to have if we want to help the community transition faster and easier towards more open research practices.

 I applaud the authors’ choice to deposit the materials in Zenodo to ensure long-term access and the choice of an open license.

 The authors take the time to explain everything in detail, but I do have two points that I would like to see tackled before final publication.

1. The part describing the FAIR qualities of the material developed doesn’t seem enough to me. For example, Table 3. (Simple steps to manage training materials following the FAIR principles) is definitely a good starting point, but FAIR is much more than that, and I believe elaborating more on this would add immense value to the work!
2. I also wonder if the authors have looked at all into accessibility features: are the files accessible to people with impaired visibility/no vision? I don’t see any HTML file, for example, which would be better to have in case screen readers were to be used.

Some other (minor) comments:

- Hyperlink in section 2 does not seem to work.
- In section 3, when referring to the TRIPLE Training Toolkit, this reference is used: Di Donato et al., 2022, which points exactly to this article I am reviewing; this seems very odd to me, perhaps an error?
- This reference comes back in the Underlying Data section, I would check that, too.

Is the case presented with sufficient detail to be useful for teaching or other practitioners?

Yes

Is the work clearly and accurately presented and does it cite the current literature?

Yes

If applicable, is the statistical analysis and its interpretation appropriate?

Not applicable

Are all the source data underlying the results available to ensure full reproducibility?

No source data required

Are the conclusions drawn adequately supported by the results?

No

Is the background of the case’s history and progression described in sufficient detail?

Yes

Reviewer Expertise:

I am an Open Science evangelist, have extensively worked on developing Open Educational Resources for Open Science. I am a full-time data scientist, versatile in FAIR and reproducible data management.

I confirm that I have read this submission and believe that I have an appropriate level of expertise to confirm that it is of an acceptable scientific standard, however I have significant reservations, as outlined above.

# Response

**Published:** 2023-05-30

**Authors:** Lottie Provost

**Affiliations:** Istituto di Linguistica Computazionale “A. Zampolli”, Consiglio Nazionale delle Ricerche, Italy

Thank you for your feedback. We have answered your comments in order of appearance below. 

 1. Thank you for your comment. Another reviewer highlighted the same concern, we therefore provide the same answer hereafter. We agree that FAIR principles are more complex than what is represented in Table 3. 

 Our aim in Table 3 is not to reduce the complexity of the concept of FAIR but rather to show beginners that following FAIR practices can also be achieved starting from small steps. We believe that FAIR can be incorporated into the organisation of open science training sessions and that our training series was designed with these principles in mind. The Table is expected to summarise good practices we applied throughout the process. We revised the article acknowledging that FAIR is a complex and multifaceted concept, clarifying that the purpose of Table 3 is to provide guidance to beginners who do not know FAIR on how to start with simple steps.

 2. Thank you for mentioning this aspect, we recognise that HTML files are needed when screen readers are used and that they improve accessibility, especially for people with impaired visibility or no vision. We have added text in the article which highlights that for improved accessibility, HTML files should be provided. 

 3. We were not able to find a broken hyperlink in Section 2. Could you please be explicit about which hyperlink you are referring to?

 ​​​​​​​​​​​​​​4. and 5. Thank you for your comments. However we have not found the reference you have mentioned. In section 3 the reference to the Triple Training Toolkit is correctly displayed, and is correctly linked in the References and in the Underlying data section. Could you please specify where you observed this reference in section 3, if the error still appears?

# Peer review: Reviewer response for version 1

**DOI:** 10.21956/openreseurope.16681.r30911

**Published:** 2023-04-13

**Authors:** Amelia Sanz

**Affiliations:** 1 Department of French Studies, Complutense University of Madrid, Madrid, Spain

This report focuses on the online training activities on Open Science delivered within the H2020 TRIPLE Project. The main goal was to address SSH research and training communities’ needs for enhanced competencies on Open Science and for stronger support in the Findable, Accessible, Interoperable, Reusable (FAIR) management of digital training materials. To do so, two reusable outputs were produced: first, a series of 12 openly accessible online training sessions specifically designed to provide skills and competencies to SSH researchers on FAIR and Open Science practices; second, the TRIPLE Training Toolkit, an open and reusable workflow containing resources for trainers (organisers and research performing organisations) to reproduce and adapt (so, to design and manage) to different training contexts in the future.

 Authors present a very good state of the art on digital skills for FAIR, Open Science and training resources. All the materials provided are a good proof of the effort they did for a common understanding of recent European Open Science advancements.

 The results presented focus on the delivery of competence-oriented training and publishing training materials as OERs: they can enable trainers in the SSH community to reproduce the case study that is showcased following best practice. All information concerning the 12 online training events held with a total of 600 attendees is available and easily findable.

 Surveys, learning objectives and learning outputs are presented in such a way that the skills and expertise required are perfectly identified: those necessary for open access publications; those regarding research data and open access, data production, management, analysis/use/reuse, dissemination; finally, those useful to to act beyond their own scholarly and disciplinary community.

 All the outputs are available in open platforms, as an example of how to promote good practices in Open Science. It is precisely the case of the sustainability document dedicated to the design and delivery of online training materials and the protocol for the collection, use and storage of related data.

 Without any doubt, an extraordinary bundle of experiences to be reused by the researchers community.

Is the case presented with sufficient detail to be useful for teaching or other practitioners?

Yes

Is the work clearly and accurately presented and does it cite the current literature?

Yes

If applicable, is the statistical analysis and its interpretation appropriate?

Yes

Are all the source data underlying the results available to ensure full reproducibility?

Yes

Are the conclusions drawn adequately supported by the results?

Yes

Is the background of the case’s history and progression described in sufficient detail?

Yes

Reviewer Expertise:

Digital Humanities, Literary Studies, E-learning

I confirm that I have read this submission and believe that I have an appropriate level of expertise to confirm that it is of an acceptable scientific standard.

# Peer review: Reviewer response for version 1

**DOI:** 10.21956/openreseurope.16681.r30958

**Published:** 2023-03-27

**Authors:** Vera Ellen Heininga

**Affiliations:** 1 Department of Developmental Psychology, Groningen University, Groningen, The Netherlands

In this TRIPLE case study, researchers and training organisers from the Social Sciences and Humanities are provided with: 1) Open Educational Resources for Open Science competence-oriented training sessions and 2) the TRIPLE Training Toolkit (i.e., guidelines for organising training events).

 I am impressed about how thoroughly the authors organized their trainings. For example, the topics for the trainings were selected based on a survey, and a short post training survey was handed out at the end of each training session to measure its impact. The results were very positive (e.g., more than 90% enjoyed the sessions). All materials were licensed (CC-BY-4.0) shared on Zenodo and NAKALA. However, I think there is also some room for improvement. I formulated my three concerns below.

 First, I doubt whether this article is really helpful for researchers. Maybe such materials are handy for researchers who study meta-science, and who thus teach about open science, but that is not necessarily a SSH field. Instead of open science training organizers and researchers I would rather say that the article is relevant for starting/beginners open science training organizers (or just: early career open science trainers) and support staff. Indeed, many universities now want to offer training on open science but support staff is often ill equipped. This would really help them out, and also help them to organise trainings without having to rely so heavily on the knowledge of researchers who volunteer to help out with such trainings (e.g., events are now often organised coming from bottom-up communities or initiatives).

 Second, the authors mention an online calendar of trainings, but I do not see how that is helpful. The materials are, but I find it difficult to see how a calendar of events that have already been organized is helpful to others. Could the authors explain maybe what they mean by the online calendar of training sessions?

 Third, the authors claim that their training series is FAIR by design, but they were less thorough on the FAIR part. For example, Table 3.  is really simple. FAIR is so much. more than how they specify it in their table.

Is the case presented with sufficient detail to be useful for teaching or other practitioners?

Yes

Is the work clearly and accurately presented and does it cite the current literature?

Yes

If applicable, is the statistical analysis and its interpretation appropriate?

Not applicable

Are all the source data underlying the results available to ensure full reproducibility?

No source data required

Are the conclusions drawn adequately supported by the results?

Yes

Is the background of the case’s history and progression described in sufficient detail?

Yes

Reviewer Expertise:

I am an open science programmeleader, and an assistant professor from the SSH field and practising open science since ten years

I confirm that I have read this submission and believe that I have an appropriate level of expertise to confirm that it is of an acceptable scientific standard, however I have significant reservations, as outlined above.

# Response

**Published:** 2023-05-30

**Authors:** Lottie Provost

**Affiliations:** Istituto di Linguistica Computazionale “A. Zampolli”, Consiglio Nazionale delle Ricerche, Italy

Thank you for your feedback. We reply to the issues you have raised in order of appearance. 

1. Thank you for your comment. We agree that the article may be more relevant for beginners in open science training and support staff than for researchers. We chose to include our case study in the Research on Research Gateway because we believe researchers who study meta-research may find the materials useful. However, the article does indeed primarily intend to provide guidance to those who want to organise open science training but do not know where to start because of a lack of experience or resources. We revised the article to clarify this point and make it more relevant for our target audience. 
2. Regarding the online calendar of training events, we were not able to find such an occurrence in the article. Could you please be explicit about which part of the article you are referring to? We have provided two possible explanations below to clarify the use of calendar tools to organise theTRIPLE training series. 
  (1) An internal calendar was used by team members to organise upcoming training sessions and share the tasks, it was not made public as it served for internal planning purposes only. By mentioning it, we wished to emphasise the importance of iterative communication and alignment of the training sessions with the project participants’ preferences and timeline to make sure that the training events indeed met their needs. 
  (2) Each training event was promoted online on a dedicated page of the website along with the date, time, location, and topic of the event. Once the event was over, the event was pushed to the bottom of the page under ‘Past Events’. The page still exists ( https://project.gotriple.eu/training/) to make the training materials available, however it is not used as an online calendar of training sessions, as it is clearly mentioned that the training series is over.
3. Thank you, we acknowledge that our discussion of FAIR in Table 3 is simplified and agree with your comment that FAIR is much more. Our aim in Table 3 is not to reduce the complexity of the concept of FAIR but rather to show beginners that following FAIR practices can also be achieved starting from small steps. We believe that FAIR can be incorporated into the organisation of open science training sessions and that our training series was designed with these principles in mind. The Table is expected to summarise good practices we applied throughout the process. We revised the article acknowledging that FAIR is a complex and multifaceted concept, clarifying that the purpose of Table 3 is to provide guidance to beginners who do not know FAIR on how to start with simple steps.
